# Global metrics on ocular biometry: representative averages and standard deviations across ten countries from four continents

**DOI:** 10.1038/s41433-022-01961-3

**Published:** 2022-02-21

**Authors:** Durga Ganesh, Shawn R. Lin

**Affiliations:** Stein Eye Institute, University of California, Los Angeles, Los Angeles, CA USA

**Keywords:** Physiology, Visual system

## Abstract

**Background/Objectives:**

We provide global averages and standard deviations for ocular biometry—axial length (AL), corneal radius of curvature (CR), anterior chamber depth (ACD), lens thickness (LT), white to white (WTW), and central corneal thickness (CT). We hope a better understanding of normal and abnormal values will help clinicians gain further insight into their surgical outcomes, especially for off-target eyes.

**Subjects/Methods:**

We searched the MEDLINE database using keywords “axial length, corneal power, anterior chamber depth, lens thickness, white to white, and corneal thickness.” We included studies that reported averages and standard deviations on eye biometry for at least 1300 eyes. Global weighted averages and standard deviations were calculated using the Cochrane method.

**Results:**

Fourteen studies were included, originating from Asia (Japan, Singapore, Myanmar, Iran, South Korea, China), Europe (Germany, United Kingdom, Portugal), Australia, and North America (United States). Global ocular biometry metrics were: AL—23.49 mm ± 1.35 mm, CR—7.69 mm ± 0.28 mm, ACD—3.10 mm ± 0.47 mm, WTW—11.80 mm ± 0.42 mm, LT—4.37 mm ± 0.43 mm, and CT—544 μm ± 38 μm. Total eyes per value ranged from 19,538 to 90,814.

**Conclusions:**

We report global ocular biometry averages and standard deviations. No eyes were from studies in Africa or South America, highlighting the need to publish eye biometry data from these continents. We hope that promoting a deeper understanding of biometry values will help clinicians gain insight into surgical outcomes and drive innovations in lens calculations.

## Introduction

Although there have been studies in the past decade that detail global ocular metrics, including this study [1] that reported average AL, ACD, and LT for 212,000 eyes stratified by sex, there remains a gap in publishing global averages in conjunction with associated standard deviations for global ocular metrics. Calculating and providing these values would allow ophthalmologists to understand their patients’ eye biometry values in the context of global values. Thus, physicians can understand how normal or abnormal these parameters may be for their individual patients.

## Methods

### Biometry data

We searched the MEDLINE database via PubMed using the keywords “axial length, corneal power, anterior chamber depth, white to white, lens thickness, and corneal thickness,” yielding 163 total papers. We included studies that reported averages and standard deviations on eye biometry for at least 1300 eyes. We also identified a study that reported ocular biometry averages for 213,000 eyes from across the world and reviewed its 35 references.

### Statistical analysis

The reported mean and standard deviations for AL, CR, ACD, WTW, LT, and CT were combined and weighted by study sample size using the Cochrane method [2]. For studies where only the confidence interval was reported rather than an explicit standard deviation, the standard deviation was back calculated using standard deviation = *s*qrt(*N*) × (Upper limit − Lower limit)/3.92. We used the two-sided, two sample *t*-test with unequal variance to compare eye biometry values between each study and all other studies. This allowed us to determine whether there was a significant difference in these studies. We calculated *p* values for each eye biometry parameter (AL, CR, ACD, WTW, LT, and CT) for each study. We compared each study’s average and standard deviation to the combined average and standard deviation for all other studies. As we compared a differential number of studies per biometric parameter, significance was achieved if *p* < 0.05/(number of studies compared per parameter) using the Bonferroni correction. Thus, we had the following thresholds for significance: AL—*p* < 0.00357, CR—*p* < 0.004, ACD—*p* < 0.0038, WTW—*p* < 0.016, LT—*p* < 0.00635, and CT—*p* < 0.01. Statistical analysis was performed using Excel.

### Global population distribution calculations

We compared the proportion of the world population per continent with our aggregate global eye dataset, to report our results in context.

## Results

**Fig. 1:**
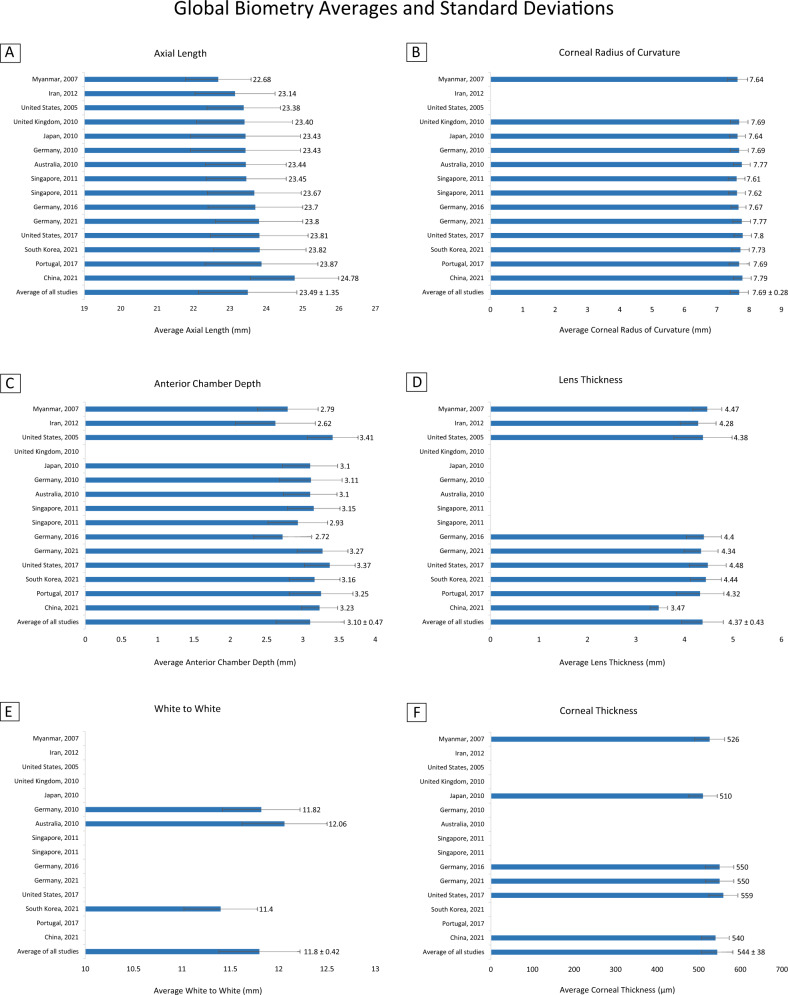
Averages and standard deviations for each study used to compute global ocular metrics. **A** Average axial length (mm), **B** corneal radius of curvature (mm), **C** anterior chamber depth (mm), **D** lens thickness (mm), **E** white to white (mm), and **F** corneal thickness (μm) reported by each study. Mean values are reported, with SD in parentheses. Studies that did not report a biometric parameter are indicated with an empty bar.

Table 1 and Fig. 1 show the averages and standard deviations for each of the studies [3–17] that were used to compute global averages and standard deviations for eye biometry, including country of origin, year of publication, and sample size. In cases where only confidence intervals were reported, we back calculated averages and standard deviations. An asterisk represents cases in which the eye biometry value for a study was significantly different from the average of the corresponding biometry values in all the other studies (e.g., AL for Portugal, 2017).

**Table 1:** Eye biometry data stratified across regions.

Country	Year	Sample size (Eyes)	AL	CR	ACD	WTW	LT	CT
China	2021	7650	24.78 mm^a^ (1.21)	7.79 mm^a^ (0.27)	3.23 mm^a^ (0.25)	Not reported	3.47 mm^a^ (0.18)	540 μm^a^ (33)
Portugal	2017	6506	23.87 mm* (1.55)	7.69 mm (0.31)	3.25 mm* (0.44)	Not reported	4.32 mm* (0.49)	Not reported
South Korea	2021	2354	23.82 mm* (1.27)	7.73 mm* (0.27)	3.16 mm* (0.35)	11.40 mm* (0.38)	4.44 mm* (0.32)	Not reported
United States	2017	4071	23.81 mm* (1.34)	7.80 mm* (0.27)	3.37 mm* (0.35)	Not reported	4.48 mm* (0.38)	559 μm* (35)
Germany	2021	5123	23.80 mm* (1.20)	7.77 mm* (0.27)	3.27 mm* (0.35)	Not reported	4.34 mm* (0.35)	550 μm* (34)
Germany	2016	5744	23.70 mm* (1.30)	7.67 mm* (0.23)	2.72 mm* (0.40)	Not reported	4.40 mm* (0.36)	550 μm* (34)
Singapore	2011	1835	23.67 mm* (1.29)	7.62 mm* (0.26)	2.93 mm* (0.41)	Not reported	Not reported	Not reported
Singapore	2011	2785	23.45 mm (1.10)	7.61 mm* (0.26)	3.15 mm* (0.36)	Not reported	Not reported	Not reported
Australia	2010	1321	23.44 mm (1.11)	7.77 mm* (0.26)	3.10 mm (0.37)	12.06 mm* (0.44)	Not reported	Not reported
Germany	2010	23,239	23.43 mm* (1.51)	7.69 mm* (0.28)	3.11 mm (0.43)	11.82 mm* (0.40)	Not reported	Not reported
Japan	2010	2838	23.43 mm* (1.51)	7.64 mm* (0.245)	3.10 mm (0.38)	Not reported	Not reported	510 μm* (34)
United Kingdom	2010	22,458	23.40 mm* (1.32)	7.69 mm* (0.27)	Not reported	Not reported	Not reported	Not reported
United States	2005	5588	23.38 mm (1.01)	Not reported	3.41 mm* (0.35)	Not reported	4.38 mm (0.60)	Not reported
Iran	2012	5190	23.14 mm* (1.10)	Not reported	2.62 mm* (0.551)	Not reported	4.28 mm* (0.368)	Not reported
Myanmar	2007	1762	22.68 mm* (0.90)	7.64 mm* (0.31)	2.79 mm* (0.42)	Not reported	4.47 mm* (0.30)	526 μm* (36)
Average of all studies	2005–2021	90,814	23.49 mm (1.35)	7.69 mm (0.28)	3.10 mm (0.47)	11.80 mm (0.42)	4.37 mm (0.43)	544 μm (38)

Mean values are reported, with SD in parentheses. Significant *p* values are indicated with an asterisk.

^a^Participants in this study were not included in the global average and standard deviation calculations as they are a non-cataract, college aged population. We provide this study’s biometry values here for reference.

The global average and standard deviation values for each eye biometry parameter are reported in Table 2. Global averages and standard deviations for AL, CR, ACD, WTW, LT, and CT were calculated from 14 studies originating from Asia (Japan, Singapore, Myanmar, Iran, South Korea), Europe (Germany, United Kingdom, Portugal), Australia, and North America (United States). Biometric parameters had the following averages with standard deviations in parentheses: AL—23.49 mm (1.35 mm), CR—7.69 mm (0.28 mm), ACD—3.10 mm (0.47 mm), WTW—11.80 mm (0.42 mm), LT—4.37 mm (0.43 mm), and CT—544 μm (38 μm). The total sample size used to calculate metrics for each biometry value ranged between 19,538 and 90,814 eyes.

**Table 2:** Global eye biometry data.

	AL	CR	ACD	WTW	LT	CT
Mean (SD)	23.49 mm (1.35)	7.69 mm (0.28)	3.10 mm (0.47)	11.80 mm (0.42)	4.37 mm (0.43)	544 μm (38)
Sample size	90,814	88,036	68,356	26,914	36,338	19,538

Mean values are reported, with SD in parentheses. The number of eyes per biometric parameter are reported as the sample size.

**Fig. 2:**
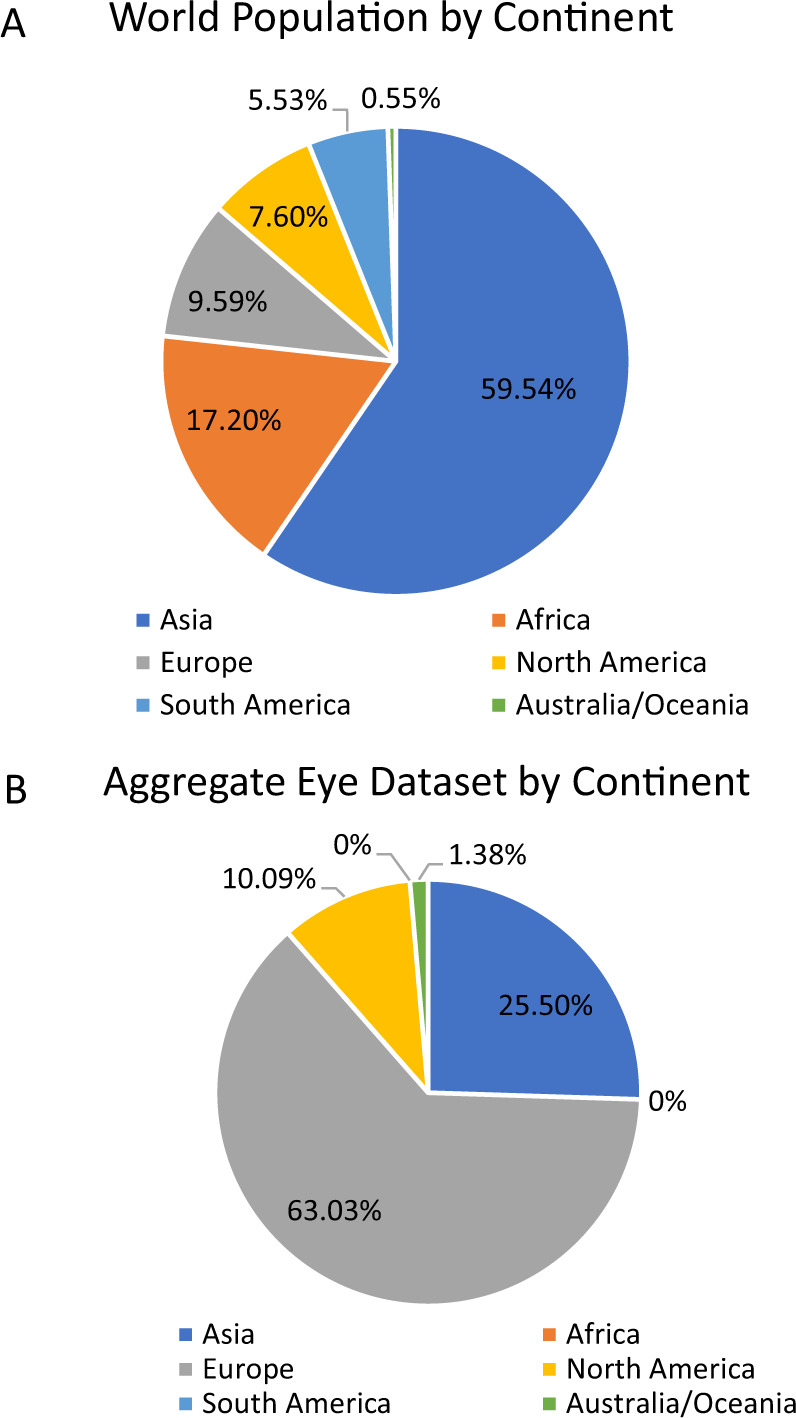
Comparison of aggregate eye dataset with the breakdown of the word population by continent. Distribution of the world population based on continent (**A**), compared to the number of individuals in our aggregate global eye dataset (**B**).

We also compared our aggregate eye dataset with the breakdown of the world population. In particular, we used a breakdown of the world population by continent [18] in 2020 to estimate the proportion of the world population that fell in the following regions: Asia, Africa, North America, South America, Europe, and Australia/Oceania (Fig. 2A). We provide a side by side comparison of these percentages, along with the breakdown of the data we used to calculate global averages (Table 3 and Fig. 2B).

**Table 3:** Distribution of the world population based on continent, compared to the number of individuals in our aggregate global eye dataset.

Continent	Population estimate (2020)	Percentage of world population (2020)	Number of individuals in aggregate eye dataset	Percentage of aggregate eye dataset	Ratio of individuals in aggregate eye dataset to world population
Asia	4,641,054,775	59.54%	16,764	25.50%	0.43
Africa	1,340,598,147	17.20%	0	0%	0
Europe	747,636,026	9.59%	45,697	63.03%	6.57
North America	592,072,212	7.60%	5588	10.09%	1.33
South America	430,759,766	5.53%	0	0%	0
Australia/Oceania	43,111,704	0.55%	1321	1.38%	2.51

## Discussion

We report large scale ocular biometry data, drawn from representative global studies across four continents. Our dataset includes eye biometry data from a diverse set of countries spanning North America, Europe, Asia, and Australia. Although our study does sample from a wide range of geographic locations, it is worth noting that the ethnic breakdown of eyes in our dataset does not match the distribution of the population of different ethnic groups (Table 3). Studies from Europe are vastly overrepresented compared to the global European population (ratio of individuals in aggregate dataset to world population: 6.57), while studies from North America (ratio of individuals in aggregate dataset to world population: 1.33) and Australia (ratio of individuals in aggregate dataset to world population: 2.51) generally match their respective population percentages. Asia is underrepresented (ratio of individuals in aggregate dataset to world population: 0.43). None of our eyes are from studies in Africa or South America, yet those two continents combined represent 22.73% of the world population. Thus, our analyses further highlight the need to collect and publish routine eye biometry data from the regions that are underrepresented and/or nonexistent in our aggregate eye dataset. We understand that data may vary according to ethnicity, so reporting data by continent has its limitations. Nonetheless, as ophthalmologists generally work within a geographic location, we feel that there is utility in reporting these values by continent, to provide clinicians with context on their patients.

Although we observed heterogeneity between eye biometry values, this does not seem to be country dependent.

In addition, the data revealed a general increase in the average AL recorded over time, as more recent studies reported longer AL values than older studies. Older studies used A scans for calculating AL, which tend to result in smaller AL. Newer studies tended to use optical low-coherence reflectometry, a technique which uses patient fixation and results in longer AL readings. Among the studies reporting lens thickness, one study [14] had a significantly different lens thickness than the other studies, as it was performed on a non-cataract, college aged population (Table 1). Thus, we have provided this study’s results as reference, without including it in our global average and standard deviation calculations for ocular biometry.

Clinicians may use our computed values for eye biometry when trying to compare their patient’s ocular biometrics to global averages. We have condensed the information in our study into a one page reference sheet, including an approximate conversion from CR to keratometry. Keratometric power (*P*_k_) was determined using *P*_k_ = (*n*_k_ − 1)*/*CR, where *n*_k_ = 1.3375 is the keratometric index of refraction and CR is in meters [19]. Our reference sheet may be easily printed for clinicians’ ease of use (Supplementary Fig. 1).

## Summary

### What was known before

- Although there have been studies in the past decade that detail global ocular metrics, including one study that reported average Axial Length, Keratometry, Anterior Chamber Depth, and Lens Thickness for 212,000 eyes stratified by sex, there remains a gap in publishing global averages in conjunction with associated standard deviations for global ocular metrics.
- Calculating and providing these values would allow for ophthalmologists to understand their patients’ eye biometry values in the context of global values, to understand how normal or abnormal these parameters may be for their individual patients.

### What this study adds

- We are the largest recent study to report large scale ocular biometry metrics, drawn from representative global studies across four continents.
- Our dataset includes eye biometry data from a diverse set of countries spanning North America, Europe, Asia, and Australia. Clinicians may use our computed values for eye biometry when trying to compare their patients’ ocular biometrics to global averages.

## Supplementary information

Supplemental Figure 1

## References

[1] Hoffer KJ, Savini G (2017). Effect of gender and race on ocular biometry. Int Ophthalmol Clin.

[2] Combine means and SDs into one group program [Internet]. StatsToDo. 2020. https://www.statstodo.com/CombineMeansSDs_Pgm.php.

[3] Hoffmann PC, Hütz WW (2010). Analysis of biometry and prevalence data for corneal astigmatism in 23 239 eyes. J Cataract Refractive Surg.

[4] Tomoyose E, Higa A, Sakai H, Sawaguchi S, Iwase A, Tomidokoro A (2010). Intraocular pressure and related systemic and ocular biometric factors in a population-based study in Japan: the Kumejima study. Am J Ophthalmol.

[5] Pan CW, Wong TY, Chang L, Lin XY, Lavanya R, Zheng YF (2011). Ocular biometry in an urban Indian population: the Singapore Indian Eye Study (SINDI). Investigative Ophthalmol Vis Sci.

[6] Tan CS, Chan YH, Wong TY, Gazzard G, Niti M, Ng TP (2011). Prevalence and risk factors for refractive errors and ocular biometry parameters in an elderly Asian population: the Singapore Longitudinal Aging Study (SLAS). Eye..

[7] Wu HM, Gupta A, Newland HS, Selva D, Aung T, Casson RJ (2007). Association between stature, ocular biometry and refraction in an adult population in rural Myanmar: the Meiktila eye study. Clin Exp Ophthalmol.

[8] Hashemi H, Khabazkhoob M, Miraftab M, Emamian MH, Shariati M, Abdolahinia T (2012). The distribution of axial length, anterior chamber depth, lens thickness, and vitreous chamber depth in an adult population of Shahroud, Iran. BMC Ophthalmol.

[9] Cartwright NK, Johnston RL, Jaycock PD, Tole DM, Sparrow JM (2010). The Cataract National Dataset electronic multicentre audit of 55 567 operations: when should IOLMaster biometric measurements be rechecked?. Eye..

[10] Shufelt C, Fraser-Bell S, Ying-Lai M, Torres M, Varma R (2005). Refractive error, ocular biometry, and lens opalescence in an adult population: the Los Angeles Latino Eye Study. Investigative Ophthalmol Vis Sci.

[11] Fotedar R, Wang JJ, Burlutsky G, Morgan IG, Rose K, Wong TY (2010). Distribution of axial length and ocular biometry measured using partial coherence laser interferometry (IOL Master) in an older white population. Ophthalmology..

[12] Kim B, Choi A, Park JH, Jeon S (2021). Prevalence of epiretinal membrane in the phakic eyes based on spectral-domain optical coherence tomography. PLoS ONE.

[13] Schuster AK, Pfeiffer N, Nickels S, Schulz A, Höhn R, Wild PS (2016). Distribution of anterior chamber angle width and correlation with age, refraction, and anterior chamber depth—the Gutenberg Health Study. Investigative Ophthalmol Vis Sci.

[14] Sun Y, Wei S, Li S, Cao K, Hu J, Yang X (2021). Distribution of ocular biometry in young Chinese eyes: the Anyang University Students Eye Study. Acta Ophthalmol.

[15] Ferreira TB, Hoffer KJ, Ribeiro F, Ribeiro P, O’Neill JG (2017). Ocular biometric measurements in cataract surgery candidates in Portugal. PLoS ONE.

[16] Fieß A, Nickels S, Schulz A, Münzel T, Wild PS, Beutel ME (2021). The relationship of ocular geometry with refractive error in normal and low birth weight adults. J Optom.

[17] Richter GM, Wang M, Jiang X, Wu S, Wang D, Torres M, Chinese American Eye Study Group, (2017). Ocular determinants of refractive error and its age-and sex-related variations in the Chinese American eye study. JAMA Ophthalmol.

[18] 7 Continents of the World—Worldometers [Internet]. Worldometers.info. 2019. https://www.worldometers.info/geography/7-continents/.

[19] Piñero DP, Camps VJ, Caravaca-Arens E, de Fez D, Blanes-Mompó FJ (2017). Algorithm for correcting the keratometric error in the estimation of the corneal power in keratoconus eyes after accelerated corneal collagen crosslinking. J Ophthalmol.

