# Supplementary material for: Global metrics on ocular biometry: representative averages and standard deviations across ten countries from four continents
Source: Eye (Lond). 2022 Feb 21;37(3):511–5. doi: 10.1038/s41433-022-01961-3 (PMC9905050; doi:10.1038/s41433-022-01961-3)
Supplement: Supplementary file 1 — Supplemental Figure 1 [file 41433_2022_1961_MOESM1_ESM.pptx]

## Slide 1
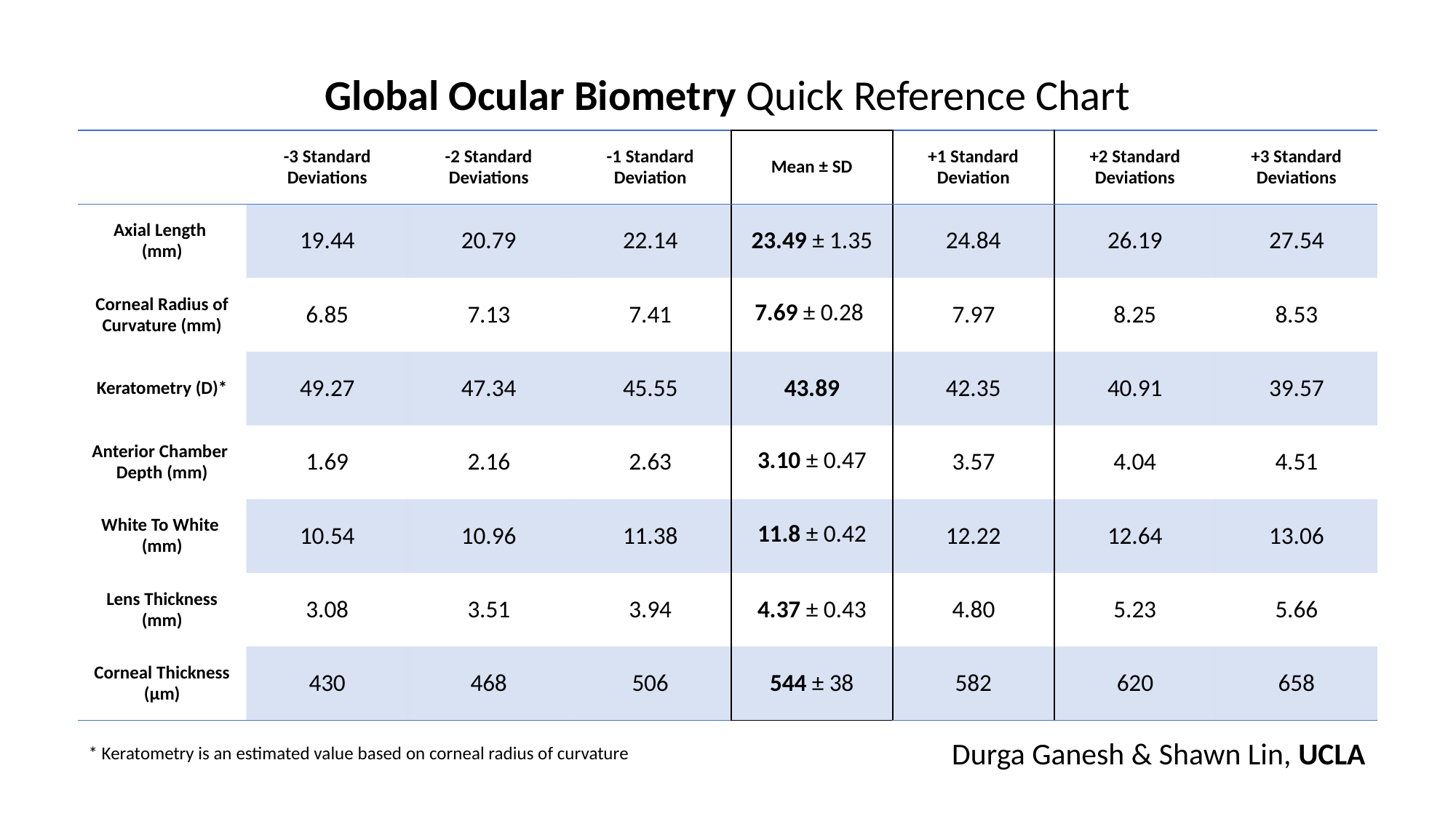

Global Ocular Biometry Quick Reference Chart
| | -3 Standard Deviations | -2 Standard Deviations | -1 Standard Deviation | Mean ± SD | +1 Standard Deviation | +2 Standard Deviations | +3 Standard Deviations |
| --- | --- | --- | --- | --- | --- | --- | --- |
| Axial Length (mm) | 19.44 | 20.79 | 22.14 | 23.49 ± 1.35 | 24.84 | 26.19 | 27.54 |
| Corneal Radius of Curvature (mm) | 6.85 | 7.13 | 7.41 | 7.69 ± 0.28 | 7.97 | 8.25 | 8.53 |
| Keratometry (D)\* | 49.27 | 47.34 | 45.55 | 43.89 | 42.35 | 40.91 | 39.57 |
| Anterior Chamber Depth (mm) | 1.69 | 2.16 | 2.63 | 3.10 ± 0.47 | 3.57 | 4.04 | 4.51 |
| White To White (mm) | 10.54 | 10.96 | 11.38 | 11.8 ± 0.42 | 12.22 | 12.64 | 13.06 |
| Lens Thickness (mm) | 3.08 | 3.51 | 3.94 | 4.37 ± 0.43 | 4.80 | 5.23 | 5.66 |
| Corneal Thickness (μm) | 430 | 468 | 506 | 544 ± 38 | 582 | 620 | 658 |
Durga Ganesh & Shawn Lin, UCLA
* Keratometry is an estimated value based on corneal radius of curvature
